# PARP1 Gene Knock-Out Increases Resistance to Retinal Degeneration without Affecting Retinal Function

**DOI:** 10.1371/journal.pone.0015495

**Published:** 2010-11-23

**Authors:** Ayse Sahaboglu, Naoyuki Tanimoto, Jasvir Kaur, Javier Sancho-Pelluz, Gesine Huber, Edda Fahl, Blanca Arango-Gonzalez, Eberhart Zrenner, Per Ekström, Hubert Löwenheim, Mathias Seeliger, François Paquet-Durand

**Affiliations:** 1 Division of Experimental Ophthalmology, Institute for Ophthalmic Research, University of Tübingen, Tübingen, Germany; 2 Ocular Neurodegeneration Research Group, Centre for Ophthalmology, Institute for Ophthalmic Research, University of Tübingen, Tübingen, Germany; 3 Department of Ophthalmology, Clinical Sciences Lund, University of Lund, Lund, Sweden; 4 Otolaryngology Department, University of Tübingen, Tübingen, Germany; Johns Hopkins, United States of America

## Abstract

Retinitis pigmentosa (RP) is a group of inherited neurodegenerative diseases affecting photoreceptors and causing blindness in humans. Previously, excessive activation of enzymes belonging to the poly-ADP-ribose polymerase (PARP) group was shown to be involved in photoreceptor degeneration in the human homologous *rd1* mouse model for RP. Since there are at least 16 different PARP isoforms, we investigated the exact relevance of the predominant isoform - PARP1 - for photoreceptor cell death using PARP1 knock-out (KO) mice. *In vivo* and *ex vivo* morphological analysis using optic coherence tomography (OCT) and conventional histology revealed no major alterations of retinal phenotype when compared to wild-type (*wt*). Likewise, retinal function as assessed by electroretinography (ERG) was normal in PARP1 KO animals. We then used retinal explant cultures derived from *wt*, *rd1*, and PARP1 KO animals to test their susceptibility to chemically induced photoreceptor degeneration. Since photoreceptor degeneration in the *rd1* retina is triggered by a loss-of-function in phosphodiesterase-6 (PDE6), we used selective PDE6 inhibition to emulate the *rd1* situation on non-*rd1* genotypes. While *wt* retina subjected to PDE6 inhibition showed massive photoreceptor degeneration comparable to *rd1* retina, in the PARP1 KO situation, cell death was robustly reduced. Together, these findings demonstrate that PARP1 activity is in principle dispensable for normal retinal function, but is of major importance for photoreceptor degeneration under pathological conditions. Moreover, our results suggest that PARP dependent cell death or PARthanatos may play a major role in retinal degeneration and highlight the possibility to use specific PARP inhibitors for the treatment of RP.

## Introduction

Blindness is a devastating condition that severely affects the quality of human life. Retinitis pigmentosa (RP) is a group of inherited neurodegenerative diseases that result in selective cell death of photoreceptors and is regarded as the main cause of blindness among the working age population in the developed world [1]. Many of the genetic mutations causing RP have been identified in recent years (for a recent list see RETNET webpage: www.sph.uth.tmc.edu/retnet) but, nevertheless, the precise mechanisms eventually causing cell death remain unknown and to date no adequate treatment for RP is available [2].

The retinal degeneration 1 (*rd1* or rd) human homologous mouse model for RP is characterized by a loss-of-function mutation in the gene encoding for the β-subunit of rod photoreceptor cGMP phosphodiesterase 6 (PDE6) [3]. The *rd1* mouse is considered a relevant model for human RP, since about 4–5% of patients are suffering from mutations in the PDE6 beta gene [4]. Non-functional PDE6 leads to accumulation of cGMP which occupies a key role in the vertebrate phototransduction cascade; however, excessively high cGMP levels trigger photoreceptor degeneration [5], [6]. The *rd1* mouse is one of the most studied animal models for RP and previously we demonstrated an involvement of excessive poly (ADP-ribose) polymerase (PARP) activity in *rd1* photoreceptor cell death [7].

PARP enzymes use NAD^+^ as a substrate to transfer ADP-ribose onto acceptor proteins [8], [9]. There are at least 16 different PARP isoforms among which PARP1 - one of the most abundant nuclear enzymes - appears to be responsible for most of the cellular poly (ADP-ribosy)lation activity [10]. PARP1 is activated by DNA strand breaks and facilitates the DNA repair process [11], [12]. On the other hand, over-activation of PARP may lead to cell death and PARP has been proposed to be a major constituent of a novel cell death mechanism termed PARthanatos [13], [14]. Accordingly, pharmacological inhibition of PARP was shown to increase cellular viability in a number of experimental systems and particularly so in the context of neurodegenerative diseases [11], [15]. Similarly, PARP inhibition protected *rd1* mouse photoreceptors [7]. Notably, though, the question which PARP isoform precisely was most important for the degeneration of photoreceptors remained open, which prevents the full understanding of the pathology.

Here, we examined the phenotype of PARP1 KO retina *in vivo*, *ex vivo* and *in vitro*. While, the retina of PARP1 KO animals appeared essentially normal in terms of morphology and function, photoreceptor cell death was greatly decreased under a specific stress paradigm that mimics inherited retinal degeneration. These results, for the first time, attribute an important role in photoreceptor cell death to PARP1 specifically and emphasize its importance for future treatments of RP.

## Results

### Comparative analysis of PARP1 KO retinal morphology and function

**Figure 1:**
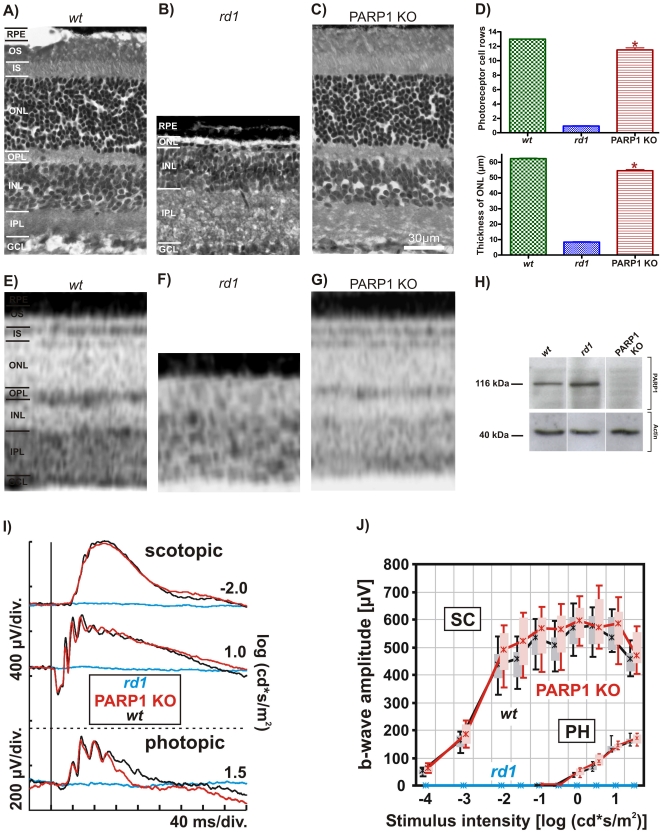
Histological and functional analysis of PARP1 KO retina. Haematoxylin/eosin staining at PN30 revealed normal morphology and layering of *wt* (**A**) retina, while in the *rd1* situation (**B**) the ONL had almost completely disappeared. In contrast, PARP1 KO retinae (**C**) appeared essentially normal, although direct comparisons with *wt* showed lower PARP1 KO values for ONL thickness and number of photoreceptor rows (quantification in **D**). SD-OCT *in vivo* imaging of *wt* (**E**), *rd1* (**F**), and PARP1 KO (**G**) retinae showed a similar picture, with PARP1 KO retina appearing slightly thinner than its *wt* counterpart. Absence of the 116 kDa PARP1 protein was confirmed using western blot (**H**). In spite of the subtle morphological changes seen in PARP1 KO, functional *in vivo* analysis using ERG under both scotopic and photopic conditions in 5 weeks old animals did not detect any differences between PARP1 KO (red traces) and *wt* control (black traces). In *rd1* animals (blue traces), however, retinal function was essentially abolished. Representative single flash ERG recordings from dark-adapted (top) and light-adapted (bottom) states are shown in (**I**), while a statistical evaluation (box-and-whisker plot) of dark-adapted (scotopic; SC) and light-adapted (photopic; PH) single flash ERG b-wave amplitudes in *wt*, *rd1*, and PARP1 KO mice is shown in (**J**). Boxes indicate the 25% and 75% quantile range, whiskers indicate the 5% and 95% quantiles, and solid lines connect the medians of the data. For each of the different experimental investigations, n = 3–4 animals from each genotype were used and analyzed independently. Error bars in (**D**) represent SEM. GCL, ganglion cell layer; IPL, inner plexiform layer; INL, inner nuclear layer; OPL, outer plexiform layer; IS, inner segment; OS, outer segment; RPE, retinal pigment epithelium.

An initial comparison of *wt, rd1 and* PARP1 KO *ex vivo* retinal morphology revealed no major differences between the *wt* and PARP1 KO and genotypes at P11 (data not shown) or at P30 (Fig. 1A–C), although at this age the ONL in PARP1 KO did not completely reach the thickness of *wt* (*wt*: 62 µm ±0.3 SEM, n = 4, *rd1*: 8 µm ±0.3 SEM, n = 3, PARP1 KO: 54 µm ±0.6 SEM, n = 4; p<0.05) (Fig. 1D). The latter was also reflected in the number of ONL photoreceptor rows (*wt*: 12.7±0.3 SEM, n = 3, *rd1*: 0.9±0.1 SEM, n = 3, PARP1 KO: 11.5±0.3 SEM, n = 4; p<0.05). Consistent with these histological data, *in vivo* optic coherence tomography (OCT) examination showed an apparently normal retinal morphology and layering together with a somewhat thinner ONL in PARP1 KO (Fig. 1E–G).

Absence of the characteristic 116 kDa band in PARP1 western blot confirmed the deficiency in protein expression in PARP1 KO (Fig. 1H). To test for possible alterations in retinal function of PARP1 KO mice, single flash ERGs were recorded from PARP1 KO and *wt* control (SV129) mice under scotopic and photopic conditions at an age of 5 weeks (Fig. 1I, J). Both rod and cone photoreceptor signalling appeared to be normal in PARP1 KO, since neither type of measurement revealed any signs of impaired retinal function.

### Cell death markers in *wt, rd1* and PARP1 KO retina

**Figure 2:**
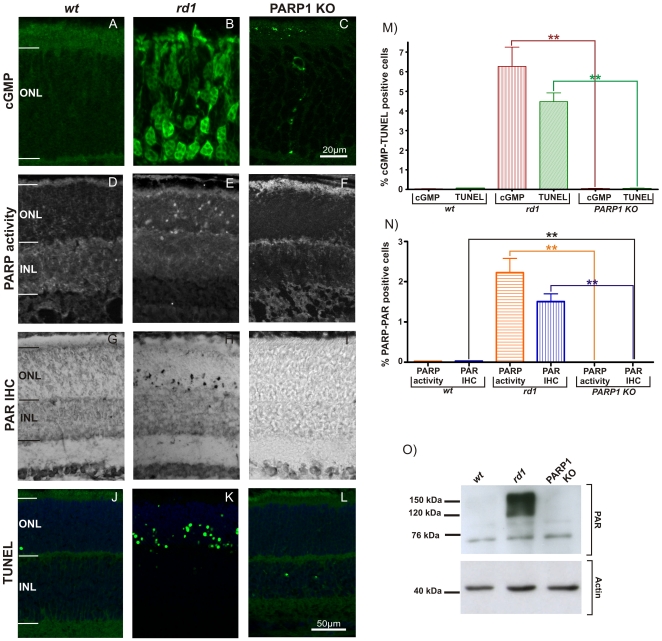
cGMP, PARP activity and TUNEL in *wt*, *rd1*, and PARP1 KO retina. At P11, immunoreactivity for cGMP was essentially absent in both *wt* and PARP1 KO retina, while in the *rd1* ONL a large number of photoreceptor cell bodies, neurites and segments were stained (**A**–**C**). The *in situ* PARP activity assay (**D–F**) and accumulation of PAR (**G**–**I**) as an indirect marker for PARP activity labeled photoreceptor nuclei only in the *rd1* situation but not in *wt* nor PARP1 KO retina. The bar graphs display the quantification of the percentages of ONL cells positive for cGMP and TUNEL (**M**), and PARP activity and PAR accumulation (**N**), respectively. Strong accumulation of PAR in *rd1* retina was confirmed using western blot (**O**). The TUNEL assay for dying cells identified large numbers of cells only in the *rd1* ONL (**J**–**L**), Retinae from n = 3–6 animals were used for each analysis and genotype. Error bars represent SEM.

In the *rd1* mouse model, retinal degeneration starts at around P11 [2] and consequently we chose this time-point for a comparative analysis of different cell death markers in *wt*, *rd1,* and PARP1 KO retina. cGMP levels were studied using immunofluorescent detection with a selective and well validated antibody [16] on *ex vivo* sections from *wt*, *rd1* and PARP KO mice at P11. While *wt* retina was essentially devoid of cGMP positive cells, many positive cells were observed in *rd1* ONL due to PDE6 dysfunction in this genotype (*wt*: 0.01% ±0.004 SEM, n = 4; *rd1*: 6.3% ±0.9 SEM, n = 4, p<0.01) (Fig. 2A, B). cGMP-positive cells were rarely seen PARP1 KO (0.004% ±0.003 SEM, n = 5) (Fig. 2C). Accumulation of cGMP corresponded to PDE6 beta expression, which was readily detectable in photoreceptor outer segments of both *wt* and PARP1 KO, but absent in the *rd1* situation (Fig. S1).

The analysis of *in situ* PARP activity at P11 (Fig. 2D–F), showed few PARP activity positive cells in the *wt* retina (0.02% ±0.008 SEM, n = 3), large numbers of PARP activity positive cells in *rd1* ONL (2.6% ±0.1 SEM, n = 3), and no detectable activity in the PARP1 KO (0.0% ±0.0 SEM, n = 3). Differences between PARP1 KO and *rd1* (p<0.01), and PARP1 KO and *wt* (p<0.05) were statistically significant.

Accumulation of poly(ADP-ribosyl)ated proteins is considered an indirect measure to confirm PARP activity. Previous studies showed that PARP activity and correspondingly accumulation of PAR were increased in *rd1* photoreceptors at P11 [7]. In the present study immunohistochemistry demonstrated very low levels of PAR accumulation in the *wt* situation (0.02% ±0.005 SEM, n = 6), high levels in *rd1* ONL (1.5% ±0.2 SEM, n = 4), and no detection in PARP1 KO (0.0% ±0.0 SEM, n = 5) (Fig. 2G–I). There were significant differences between *in vivo* PARP1 KO and *rd1* (p<0.01) as well as PARP1 KO and *wt* (p<0.05). Western blot analysis for PAR confirmed the immunohistochemical results showing a strong accumulation of high molecular weight poly(ADP-ribosyl)ated proteins only in *rd1* retinal tissue samples (Fig. 2O).

We then used the TUNEL assay to identify degenerating cells in the different genotypes. In the ONL, *wt* retinae showed only very few TUNEL positive, dying cells, while much higher numbers were detected in the *rd1* situation (*wt*: 0.1% ±0.006 SEM, n = 3; *rd1*: 4.5% ±0.4 SEM, n = 3) (Fig. 2J, K). Similar to *wt*, PARP1 KO retinae showed very low numbers of TUNEL positive cells (PARP1 KO: 0.04% ±0.01 SEM, n = 3) (Fig. 2L). Quantification and statistical analysis revealed no significant difference (p = 0.31) between *wt* and PARP1 KO but a significant difference (p<0.01) between PARP1 KO and *rd1* (Fig. 2M). In line with the histological data, these results indicated that there was no major degeneration phenotype in the ONL of PARP1 KO, which therefore, in all aspects studied here, appeared to behave like *wt*.

### PARP1 KO reduces photoreceptor cell death induced by PDE6 inhibition

**Figure 3:**
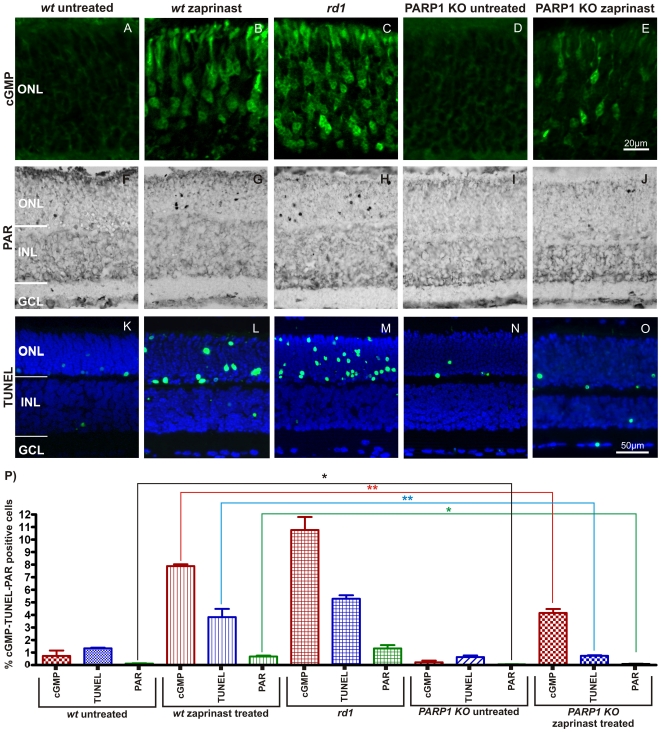
PARP1 KO animals are resistant to PDE6 inhibition induced photoreceptor cell death. Organotypic retinal explant cultures obtained from *wt* and PARP1 KO animals were treated with the PDE6 inhibitor zaprinast and compared to untreated *wt*, *rd1* and PARP1 KO cultured retinae. Control *wt* retinae at P11 *in vitro* showed minimal immunoreactivity to cGMP-antibody in the ONL (**A**). Zaprinast treated *wt* retinae exhibited strongly increased intracellular cGMP levels (**B**), similar to what was observed in PDE6 mutant *rd1* retina (**C**). While PARP1 KO did not display cGMP immunoreactivity (**D**), it responded to zaprinast treatment with a moderate, but significant, elevation of cGMP positive ONL cells (**E**). Accumulation of PAR as an indirect marker for PARP activity (**F**–**J**) was found in large amounts only in zaprinast treated *wt* or *rd1* ONL but notably absent in PARP1 KO preparations and *wt* retinae. The TUNEL assay for dying cells generally followed a similar pattern (**K**–**O**): The *rd1* mutant or PDE6 inhibition resulted in marked increases of positive cells in the ONL only. The bar graph (**P**) illustrates the quantification of the three parameters assayed. Explant cultures from n = 3–6 animals were used for each treatment situation and genotype. Error bars represent SEM.

Zaprinast is a selective PDE5/6 inhibitor [17], [18] which in a concentration dependent manner raises intracellular cGMP levels and causes cGMP-dependent photoreceptor degeneration closely resembling the *rd1* degeneration (Fig. S2) [6], [19]. Here, 100 µM zaprinast was used to mimic the *rd1* situation on *wt* and PARP1 KO retinal explants cultured between postnatal days 5–11. Successful PDE6 inhibition was confirmed by an increased cGMP immunofluorescence. While untreated *wt* retina is essentially devoid of cGMP immunoreactivity (0.7% ±0.4 SEM, n = 5), the zaprinast treated *wt* ONL showed large numbers of cGMP positive cells (7.8% ±0.1 SEM, n = 4), comparable to the *rd1* situation (10.6% ±0.7 SEM, n = 7) (Fig. 3A–C). Untreated PARP1 KO displayed very low cGMP immunoreactivity (0.2% ±0.1 SEM, n = 3), which was strongly increased by zaprinast treatment (4.2% ±0.3 SEM, n = 4) (Fig. 3D, E). The zaprinast induced increase in cGMP positivity was significantly lower in PARP1 KO when compared to *wt* (p<0.01). However, in relative terms the zaprinast induced elevation in cGMP was more pronounced in PARP1 KO (21-fold increase) than in *wt* (11-fold increase).

Immunohistochemistry for PAR illustrated few positive cells in the ONL of untreated *wt* situation (0.1% ±0.02 SEM, n = 3), larger numbers of positive cells in zaprinast treated *wt* (0.7% ±0.07 SEM, n = 5) and *rd1* ONL (1.3% ±0.3 SEM, n = 3), and very few positive cells in untreated (0.05% ±0.01 SEM, n = 3, p<0.05) and zaprinast treated PARP1 KO retinae (0.1% ±0.02 SEM, n = 3, p<0.05) (Fig. 3F–J).

The TUNEL assay for dying cells identified only very few positive cell in P11 *wt* ONL i*n vitro* (*wt*: 1.3% ±0.1 SEM, n = 3), while zaprinast treatment raised their number to levels comparable to untreated *rd1* retina (*wt* + zaprinast: 3.9% ±0.1 SEM, n = 6; *rd1*: 5.3% ±0.3 SEM, n = 5) (Fig. 3K–M). Untreated PARP1 KO retina displayed very low numbers of TUNEL positive cells (0.6% ±0.1 SEM, n = 3) similar to untreated *wt*. Importantly, zaprinast treatment resulted only in a minor elevation of cell death in PARP1 KO retina when compared to zaprinast treated *wt* (PARP1 KO + zaprinast: 0.7% ±0.04 SEM, n = 5, p<0.01) (Fig. 3N, O). In relative terms, zaprinast treatment resulted in a 200% elevation of cell death in *wt* retina, compared to only 17% increase in PARP1 KO (Fig. 3P).

Together, these results suggest that the photoreceptor cell death that follows upon PDE6 inhibition and subsequent accumulation of cGMP, to a major extent is dependent on PARP1 activity, since PARP1 KO displayed strong resistance to this paradigm of induced photoreceptor degeneration.

## Discussion

Cell death, in particular in the context of neurodegenerative diseases, has frequently been found to be associated with excessive PARP activity [10], [11] and we have previously found strong PARP activation to be causally connected to photoreceptor cell death [7]. Nevertheless, at the beginning of this study it was not clear which one of the 16 different PARP isoforms might be responsible for this detrimental effect. Here, we show that PARP activity during photoreceptor neurodegeneration is caused to a major extent by the PARP1 isoform specifically. While *wt* photoreceptors were highly susceptible to a stress paradigm mimicking the *rd1* type of inherited retinal degeneration, PARP1 KO photoreceptors were resistant to such stress.

### PARP activity in cellular physiology

PARP enzymes play ambiguous roles in cellular physiology. They are important mediators of DNA repair and strongly protect cells against genotoxic stressors [20], [21], notably because poly(ADP-ribosyl)ation of DNA associated histones causes relaxation of the chromatin structure, allowing DNA repair enzymes to access the site of the strand break, thereby facilitating the DNA repair process [10], [12]. On the other hand, an excessive PARP activation may overstrain the cellular metabolism, leading to an energetic collapse and eventually cell death [22], [23]. In this respect excessive consumption of the PARP substrate NAD^+^ seems to be of particular importance, since this will indirectly result in depletion of cellular ATP [10], [22].

In conjunction with its antagonist poly-ADP-ribose-glycohydrolase (PARG), PARP activity may result in the generation of free PAR polymers. These may cause release of a mitochondrial protein termed apoptosis inducing factor (AIF) resulting in its translocation to the nucleus, widespread DNA fragmentation and cell death [24], as seen in PARP dependent cell death of primary cortical neurons [25]. Alternatively, free PAR may act on transient-receptor-potential (TRP) ion channels causing excessive calcium influx [26], [27] with potential repercussions on calcium-dependent calpain-type protease activation and therefore photoreceptor cell death [28]. Interestingly, auditory receptor cell death following acoustic trauma and cochlear ischemia has also been connected with excessive PARP activation, suggesting that sensory cells might be particularly susceptible to undergo PARP dependent cell death [29], [30].

PARP1 KO mice develop normally and show no particular phenotype. However, these animals are susceptible to developing epidermal hyperplasia and obesity at older ages [31] and cells lacking PARP1 are susceptible to genotoxic stress [21]. Future investigations will have to determine whether reduced DNA repair capabilities, or other PARP related actions, are responsible for the slight decrease in retinal thickness observed in PARP1 KO animals. Interestingly, PARP1 KO animals display increased resistance to streptozotocin induced cell death of pancreatic beta cells [32], ischaemic brain injury [33], and ocular deprivation induced cell death in the lateral geniculate nucleus [34]. Indeed, PARP is supposed to play a central role in a novel form of caspase-independent cell death which involves excessive activation of PARP, formation of poly-ADP-ribose polymers, translocation of AIF protein from mitochondria to the nucleus, chromatin condensation, and large DNA fragmentation. Because of its dependence on PARP activity and the generation of PAR polymers this cell death mechanism was tentatively termed PARthanatos [13], [35]. Taken together, PARP1, apart from its functions during normal cell physiology appears to be of major importance for cell death, particularly in neuronal cells.

### PARP and photoreceptor cell death mechanisms

It has previously been shown that photoreceptor cell death in the *rd1* mouse is associated with nuclear translocation of AIF [36] and oxidative DNA damage, factors which both colocalizes with PARP activity in photoreceptor nuclei [7]. Our finding that PARP1 KO photoreceptors are highly resistant to PDE6 inhibition induced degeneration confirms these previous results and points to the PARP1 isoform as a major contributor to PARP dependent photoreceptor cell death. However, while PARP1 KO dramatically reduced the accumulation of PAR in all situations tested, PAR accumulation was not abolished altogether. This suggests that other PARP isoforms such as PARP2 [21], [37], [38], [39] may, to a limited extent, compensate for the lack of PARP1 expression.

Apart from PARP activity, photoreceptor cell death in the *rd1* mouse model for RP has been found to be characterized by an elevation of cGMP levels, activation of PKG, calpains and histone deacetylases (HDACs) [5], [6], [40]. The PARP activity in *rd1* photoreceptors might have a bearing on calpain activation [15] and could itself be governed by the activity of HDACs, since HDAC inhibition in *rd1* retina also abolished PARP activity [38]. Moreover, deacetylation and poly(ADP-ribose)ylation were exactly coincidental in degenerating *rd1* photoreceptors [40] also suggesting a sequential activation of first HDAC then PARP during cell death.

Even though photoreceptor degeneration has in the past often been addressed as an apoptotic process, it takes place independent of critical features of apoptosis such as *de novo* protein biosynthesis, caspase activation, or cytochrome *c* release (*reviewed in:* 2). The finding that photoreceptor degeneration induced by PDE6 inhibition was strongly reduced in PARP1 deficient animals suggests that PARthanatos or a closely related mechanism is responsible for cell death in this situation. If this was the case then in turn the wealth of knowledge available for the *rd1* degeneration might also be used to improve the understanding of this novel cell death mechanism.

### Conclusion

We have shown a causal involvement of PARP1 in a retinal photoreceptor degeneration paradigm, that mimics inherited neurodegeneration as it occurs in the human homologous *rd1* mouse model. PARP1 KO prevented photoreceptor cell death *in vitro*, a result that highlights the importance of PARP1 as a novel therapeutic target in retinal degeneration both for pharmacological and genetic treatment approaches. A reduced PARP1 activity would most likely not compromise retinal function, since absence of PARP1 does not alter retinal function. These findings also propose that photoreceptor cell death may be governed by an alternative cell death mechanism, possibly related to the recently described PARthanatos.

## Materials and Methods

### Experimental animals

Animals were housed under standard white cyclic lighting, had free access to food and water, and were used irrespective of gender. C3H *rd1* mice [41], PARP1 KO mice [31] and wild-type (*wt*) SV129 mice were used. All procedures were approved by the Tübingen University committee on animal protection (Einrichtung für Tierschutz, Tierärztlichen Dienst und Labortierkunde directed by Dr. Franz Iglauer) and performed in accordance with the ARVO statement for the use of animals in ophthalmic and visual research. Protocols compliant with §4 of the German law on animal protection were reviewed and approved by Dr. Ulf Scheurlen and Dr. Susanne Gerold (Einrichtung für Tierschutz, Tierärztlichen Dienst und Labortierkunde; Registration No.: 16/12/08-1, 10/02/10-1). Since experiments were carried out on *ex vivo* retinal explants (see below), no further permits were required. Because in the *rd1* retina critical changes are apparent at post-natal day 11 (P11) [30], [42], most comparisons were carried out at this age.

### Morphological characterization

Haematoxylin/Eosin staining was used for *ex vivo* characterization of PARP1 KO retinae. Fixed cryosectioned retinae were stained in Harris haematoxylin solution (Vector Laboratories, CA, USA, H-3401) for 3 minutes and then washed in bidestilled water for 1 minute. Following a brief, 2s exposure to 25% hydrochloric acid in ethanol, the sections were washed again and counterstained in Accustain eosin Y solution (Sigma-Aldrich, Munich, Germany, HT-110-1-16) for 30 seconds to 1 minute. The sections were dehydrated in a 70% –96% –100% alcohol series, washed in xylene for 2 minutes, and then mounted with DPX mounting medium for histology (Sigma-Aldrich).

### Electroretinographic Analysis

Electroretinograms (ERGs) were recorded binocularly according to previously described procedures [43], [44]. The ERG equipment consisted of a Ganzfeld bowl, a direct current amplifier, and a PC-based control and recording unit (Multiliner Vision; VIASYS Healthcare GmbH, Hoechberg, Germany). Mice of 5 weeks age were dark-adapted overnight and anaesthetised with ketamine (66.7 mg/kg body weight) and xylazine (11.7 mg/kg body weight). The pupils were dilated and single flash ERG responses were obtained under dark-adapted (scotopic) and light-adapted (photopic) conditions. Light adaptation was accomplished with a background illumination of 30 candela (cd) per square meter starting 10 minutes before recording. Single white-flash stimulus intensity ranged from −4 to 1.5 log cd*s/m^2^ under scotopic and from −2 to 1.5 log cd*s/m^2^ under photopic conditions, divided into 10 and 8 steps, respectively. Ten responses were averaged with an inter-stimulus interval of either five seconds or 17 seconds (for 0, 0.5, 1, and 1.5 log cd*s/m^2^).

### Spectral domain optical coherence tomography (SD-OCT)

SD-OCT imaging was performed immediately following ERG, i.e. animals remained anaesthetized. Mouse eyes were subjected to SD-OCT using the commercially available Spectralis™ HRA+OCT device from Heidelberg Engineering (Heidelberg, Germany) featuring a broadband superluminescent diode at λ = 870 nm as low coherent light source. Each two-dimensional B-Scan recorded at 30° field of view consisted of 1536 A-Scans, which were acquired at a speed of 40,000 scans per second. Optical depth resolution was approximately 7 µm with digital resolution reaching 3.5 µm [45]. The adaptation for the optical qualities of the mouse eye was described previously [46].

### Retinal explant cultures

Organotypic retinal cultures that included the retinal pigment epithelium (RPE) were prepared in principle as previously published [47]. Briefly, P5 animals were sacrificed, the eyes enucleated and pretreated with 12% proteinase K (ICN Biomedicals Inc., OH, USA; 193504) for 15 minutes at 37°C in R16 serum free culture medium (Invitrogen Life Technologies, Paisley, UK; 07490743A). Proteinase K was blocked by addition of 10% fetal bovine serum, followed by rinsing in serum-free medium. In the following, cornea, lens, sclera and choroid were removed carefully, with only the RPE remaining attached to the retina. The explant was then cut into four wedges to give a clover-leaf like structure which was transferred to a culture membrane insert (Millipore AB, Solna, Sweden; PIHA03050) with the RPE facing the membrane. The membrane inserts were placed into six well culture plates and 1.4 ml of R16 medium with supplements [47] was added. The cultures were incubated at 37°C in a humidified 5% CO_2_ incubator. The culture medium was changed every 2 days during 6 culturing days. Retinal explants were left without treatment for 2 days (until P7), followed by zaprinast (100 or 200 µM; Sigma Z0878) treatment. Zaprinast was prepared in dimethyl sulfoxide (DMSO; Sigma D2650) and diluted in R16 serum free culture medium with supplements. For controls, the same amount of DMSO was diluted in culture medium.

### TUNEL Assay

The terminal deoxynucleotidyl transferase dUTP nick end labeling (TUNEL) assay was performed on cryosections from treated/untreated *wt*, PARP1 KO, and *rd1* retinae, using an *in situ* cell death detection kit conjugated with fluorescein isothiocyanate (Roche Diagnostics, Mannheim, Germany). For controls, terminal deoxynucleotidyl transferase enzyme was either omitted from the labelling solution (negative control), or sections were pre-treated for 30 min with DNAse I (Roche, 3 U/ml) in 50 mM Tris-HCl, pH 7.5, 1 mg/ml BSA to induce DNA strand breaks (positive control). Negative control gave no staining at all, while positive control stained all nuclei in all layers of the retina (not shown, *see:* 7).

### Immunostaining

4% PFA fixed, frozen retinal sections from P11 animals or cultured retinae, were dried for 30–60 minutes at 37°C. Subsequently, the tissue was rehydrated in PBS, and pre-incubated for 1 hour at RT in blocking solution, containing 10% normal serum, and 0.1% or 0.3% Triton in PBS (PBST). Immunohistochemistry was performed overnight at 4°C, using primary antibodies directed against cGMP (obtained from Jan de Vente, Maastricht University; *see:* 16; dilution 1∶500), PAR (Alexis Biochemicals, Lörrach, Germany; dilution 1∶200; Order No.: 804-220), and PDE6 beta (Affinity Bioreagents; dilution 1∶400; Order No.: PA1-722) diluted in blocking solution. The tissue was rinsed in PBST, and incubated for 1 hour with Alexa 488 conjugated secondary antibody, (1∶200-1∶750, Invitrogen), diluted in PBST. Sections were rinsed in PBS, and mounted in Vectashield with DAPI (Vector, Burlingame, CA, USA).

### Western blot (WB)

Retinal tissue from PARP1 KO, *wt* and *rd1* mice were homogenized in buffer as described previously [7] with a Heidolph DIAX 600 homogenizer (Heidolph, Schwabach, Germany) or a manual homogenizer (glass to glass). Bradford assay was used for determination of protein concentration. For separation of proteins, SDS-PAGE 10–12% gradient gel (at 55 V) was used and 27 µg protein was loaded per well. Subsequently, the proteins were transferred to PVDF membranes (GE Healthcare, UK). Membranes were blocked in Roti block (Roth, Karlsruhe, Germany) blocking buffer for 1 hour at room temperature (RT). Membranes were incubated in primary antibodies against PARP1 (BD Pharmingen, Heidelberg, Germany; 556362), PAR (see above), actin (Sigma-Aldrich; A 2668) at a dilution of 1∶1000 in buffer containing PBST and 5% dried milk (Roth) overnight at 4°C. Membranes were washed with PBST and incubated with horseradish peroxidase conjugated secondary antibody (GE Healthcare, UK) for 1 hour at RT. Hyperfilm (GE Healthcare, UK) detection system was used as a membrane developer. Films were scanned and quantified using ImageJ (National Institutes of Health, Washington, USA).

### PARP activity assay

Eyes from PARP1 KO, *wt* and *rd1* mice were enucleated, frozen immediately on dry ice (−72°C), followed by cryosectioning. A biotin-avidin blocking kit (Vector) was used to block endogenous biotin and to reduce background. After incubation with PARP reaction mixture (10 mM MgCl_2_, 1 mM dithiothreitol, 5 µM biotinylated NAD (Trevigen, Gaithersburg, MD, USA) in 100 mM Tris buffer with 0.2% Triton X100, pH 8.0) for 2.5 h at 37°C, the sections were washed with PBS, 3 times for 5 minutes. The biotin incorporated by PARP activity was then detected by fluorescently labeled avidin (1∶800 in PBS, 1 h at RT). After 3 times 5 min washing in PBS, the sections were mounted in Vectashield (Vector). For controls, biotinylated-NAD^+^ was omitted from the reaction mixture resulting in absence of detectable reaction product.

### Microscopy, Cell counting, and Statistics

Microscopy was performed using a Zeiss Imager Z1 Apotome Microscope. Images were taken with a Zeiss Axiocam digital camera, using Zeiss Axiovision 4.7 software. Image enhancements (Contrast, Colors) were done in paired fashion using Corel Draw X3 software.

The percentages of ONL cells positive in the different assays (PARP activity, PAR IHC, TUNEL) were assessed and calculated in a blinded fashion as reported previously [6], [28]. For each animal the central areas (in proximity to the optic nerve) of at least 3 sections were quantified to yield an average value, and at least 3 different animals were analyzed for each time-point and genotype. Values are given as mean ± standard error of the mean (SEM).

Statistical analysis was performed using GraphPad Prism 4.01 software (GraphPad Software, La Jolla, CA, USA) and two-tailed Student's *t* test. Levels of significance were: *  =  p<0.05, **  =  p<0.01, ***  =  p<0.001.

## Supporting Information

Figure S1 **PDE6 beta expression in *wt*, *rd1*, and PARP1 KO retinae.** At PN11, immunostaining revealed PDE6 beta expression (green) in photoreceptor outer segments of *wt* retina, while PDE6 beta protein was undetectable in *rd1* retina. In PARP1 KO retina PDE6 beta expression followed the pattern of *wt*. DAPI (blue) was used as nuclear counterstain. The images shown are representative for immunostainings performed on retinal cross-sections from at least 3 different animals for each genotype.

Figure S2 **Zaprinast treatment induces cGMP accumulation and cell death in a concentration dependent manner.** Untreated organotypic retinal cultures derived from *wt* animals showed very few cGMP (**A**) and TUNEL (**D**) positive cells at P11 *in vitro*. PDE6 inhibition with zaprinast caused cGMP accumulation (**B**, **C**) and cell death (**E**, **F**) that increased together with zaprinast concentration (Quantification in **G**). Explant cultures from n=3-8 *wt* animals were used for each treatment situation. Error bars represent SEM.
