# Supplementary figures and images for: PARP1 Gene Knock-Out Increases Resistance to Retinal Degeneration without Affecting Retinal Function
Source: PLoS One. 2010 Nov 23;5(11):e15495. doi: 10.1371/journal.pone.0015495 (PMC2990765; doi:10.1371/journal.pone.0015495)

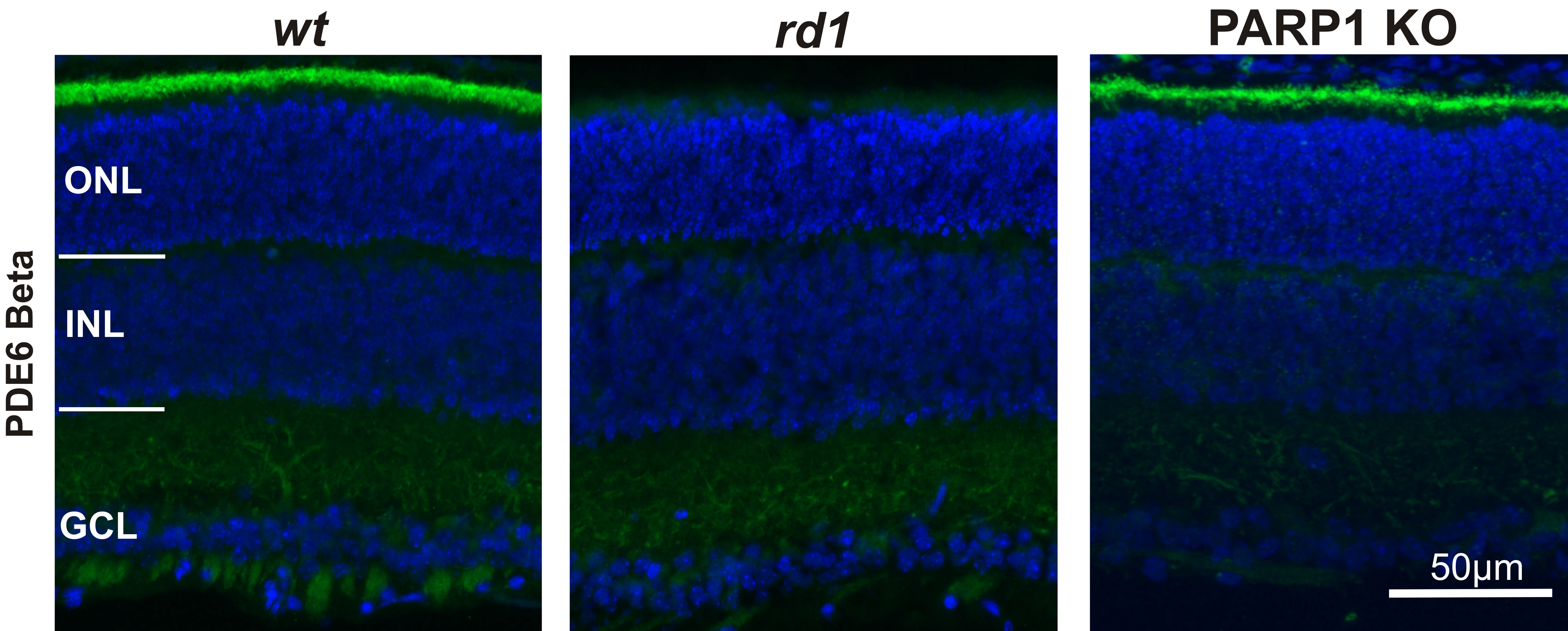

Supplement: Figure S1 — PDE6 beta expression in wt, rd1, and PARP1 KO retinae. At PN11, immunostaining revealed PDE6 beta expression (green) in photoreceptor outer segments of wt retina, while PDE6 beta protein was undetectable in rd1 retina. In PARP1 KO retina PDE6 beta expression followed the pattern of wt. DAPI (blue) was used as nuclear counterstain. The images shown are representative for immunostainings performed on retinal cross-sections from at least 3 different animals for each genotype. [file pone.0015495.s001.tif]

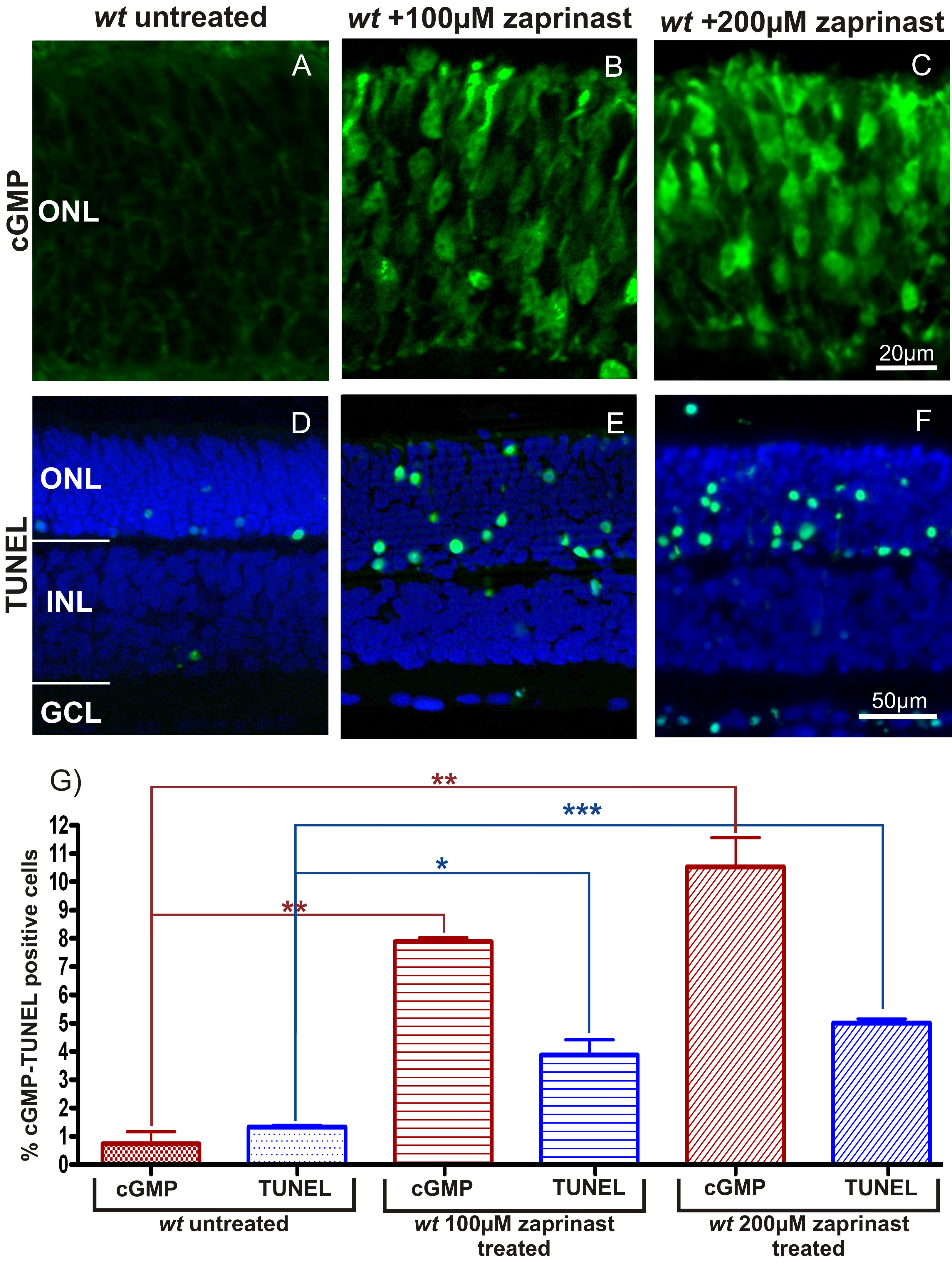

Supplement: Figure S2 — Zaprinast treatment induces cGMP accumulation and cell death in a concentration dependent manner. Untreated organotypic retinal cultures derived from wt animals showed very few cGMP (A) and TUNEL (D) positive cells at P11 in vitro. PDE6 inhibition with zaprinast caused cGMP accumulation (B, C) and cell death (E, F) that increased together with zaprinast concentration (Quantification in G). Explant cultures from n=3-8 wt animals were used for each treatment situation. Error bars represent SEM. [file pone.0015495.s002.tif]
